# Antioxidant Activity and Anthocyanin Contents in Olives (*cv* Cellina di Nardò) during Ripening and after Fermentation

**DOI:** 10.3390/antiox8050138

**Published:** 2019-05-18

**Authors:** Alessio Aprile, Carmine Negro, Erika Sabella, Andrea Luvisi, Francesca Nicolì, Eliana Nutricati, Marzia Vergine, Antonio Miceli, Federica Blando, Luigi De Bellis

**Affiliations:** 1 Department of Biological and Environmental Sciences and Technologies (DiSTeBA), Salento University, Via Prov. le Lecce-Monteroni, 73100 Lecce, Italy; alessio.aprile@unisalento.it (A.A.); carmine.negro@unisalento.it (C.N.); andrea.luvisi@unisalento.it (A.L.); francesca.nicoli@unisalento.it (F.N.); eliana.nutricati@unisalento.it (E.N.); marzia.vergine@unisalento.it (M.V.); antonio.miceli@unisalento.it (A.M.); luigi.debellis@unisalento.it (L.D.B.); 2 Institute of Sciences of Food Production (ISPA), National Research Council (CNR), Research Unit of Lecce, Via Prov. le Lecce-Monteroni, 73100 Lecce, Italy; federica.blando@ispa.cnr.it

**Keywords:** olive, *Olea europaea*, anthocyanin, cyanidin 3-glucoside, cyanidin 3-rutinoside, oxygen radical absorbance capacity (ORAC), high performance liquid chromatography mass spectrometry (HPLC-MS).

## Abstract

The olive tree “Cellina di Nardò” (CdN) is one of the most widespread cultivars in Southern Italy, mainly grown in the Provinces of Lecce, Taranto, and Brindisi over a total of about 60,000 hectares. Although this cultivar is mainly used for oil production, the drupes are also suitable and potentially marketable as table olives. When used for this purpose, olives are harvested after complete maturation, which gives to them a naturally black color due to anthocyanin accumulation. This survey reports for the first time on the total phenolic content (TPC), anthocyanin characterization, and antioxidant activity of CdN olive fruits during ripening and after fermentation. The antioxidant activity (AA) was determined using three different methods. Data showed that TPC increased during maturation, reaching values two times higher in completely ripened olives. Anthocyanins were found only in mature olives and the concentrations reached up to 5.3 g/kg dry weight. AA was determined for the four ripening stages, and was particularly high in the totally black olive fruit, in accordance with TPC and anthocyanin amounts. Moreover, the CdN olives showed a higher TPC and a greater AA compared to other black table olives produced by cultivars commonly grown for this purpose. These data demonstrate the great potential of black table CdN olives, a product that combines exceptional organoleptic properties with a remarkable antioxidant capacity.

## 1. Introduction

The olive cultivar “Cellina di Nardò” (CdN), also known as “Leccese”, “Saracena”, “Visciola”, “Asciulo”, or “Muredda”, is an Italian variety that is widespread in Salento (Apulia, South Italy), especially in the Province of Lecce, although it is also grown in the territories of Taranto and Brindisi, covering a total of about 60,000 hectares. However, since 2013, Salento has been suffering from a devastating olive disease (the olive quick decline syndrome) caused by the bacteria *Xylella fastidiosa* [1,2] that has progressively destroyed hundreds of thousands of olive trees. Unfortunately, the CdN cultivar is sensitive to *Xylella fastidiosa*, and in the coming years is likely to disappear completely from the Salento area [3,4]. For this reason, a complete characterization of such traditional cultivars is required, as many plants are still cultivated in South Italy.

The CdN tree is vigorous; the branches at the top are erect, while the lateral ones almost pendulous, and the tree can reach a height of 20 m. The leaves have an elliptical elongated shape; the upper side of the leaf is dark green while the lower one is silvery gray. The blooming stage is quite early, and the inflorescence results in 15–20 flowers. The fruit is an elliptical drupe, slightly asymmetrical, with a color ranging from green to black; when ripening is completed, it has a reduced size, with a weight ranging from 1.5 to 2.0 g, and a low oil yield (15–17%).

The drupes have a high resistance to detachment and are not suitable for mechanical harvesting. On the contrary, this cultivar is valued by local farmers due to the slow vegetative growth, good fructification in adverse conditions, and good tolerance to cold and various pests. Despite the low oil yield and difficulties in harvesting, the CdN is cultivated mainly for oil production, which is characterized by an intense fruity flavor. The oil obtained from CdN and Ogliarola olives (another traditional cultivar widespread in Salento) is guaranteed by the brand “Terra d’Otranto” as a ‘‘Protected Designation of Origin”, and presents some specific characteristics as a consequence of the geographical influence, pedoclimatic conditions, agronomic techniques, and oil processing.

The olive fruit consists of water (about 50%) and fats (20%), and the remaining part is made up of nitrogenous compounds, cellulose, sugars, and secondary metabolites [5]. The secondary metabolites of fresh fruit and fermented olives can vary greatly. In fact, the different processes of extraction and purification can modify the chemical structure of the molecules due to exposure to oxygen or solvents or even pH changes, situations that can commonly occur in phenolic metabolism [6]. The proportion of phenolic compounds within the edible part of the olive is considerable and can reach concentrations ranging from 1% to 3% of the fresh weight of the pulp [7]. There is a complex mixture of phenolic compounds in olives, some of which are present at very low concentrations and as a consequence are difficult to identify [8]. Moreover, phenolic and secondary metabolites are not uniformly present in diverse parts of the fruit: most of them are present in the pulp (about 85–90%), followed by the peel (about 8–12%), and then by the seed (1–2%).

Among phenolic compounds, oleuropein is generally the most represented among the various olive cultivars, reaching concentrations up to 140 mg/g fresh weight (FW) [9]. Oleuropein belongs to the secoiridoid family, as well as other compounds usually found in olives like dimethyl oleuropein, verbascoside, ligstroside, and nüzhenide [10]. In particular, oleuropein, dimethyl oleuropein, and verbascoside have been found in the all parts of the olive fruit (pulp, skin, and seed); conversely, the presence of nüzhenide was reported only in the seed [11]. In the olive fruit there are also flavones such as luteolin-7-glucoside, flavonols such as quercetin 3-rutinoside [12], anthocyanins like cyanidin-3-rutinoside and cyanidin-3-glucoside, and phenolic acids such as hydroxybenzoic, gallic, ferulic, caffeic, vanillic, and syringic acid [13].

All these secondary metabolites are of great interest for human health because of their antioxidant activity and properties with respect to cancer prevention, inflammatory disorders, and cardiovascular diseases [14,15].

The health properties of CdN when used as table olive have not yet been reported, and the *Xylella fastidiosa* threat suggests their urgent investigation due to the extinction risk caused by the pathogen. Therefore, in this paper a characterization of the secondary metabolites during ripening stages was carried out. Moreover, six different table olive cultivars were compared to CdN cultivars to establish the best food in terms of antioxidant effects and phenolic compounds.

## 2. Materials and Methods

### 2.1. Plant Material and Samples Preparation

**Figure 1:**
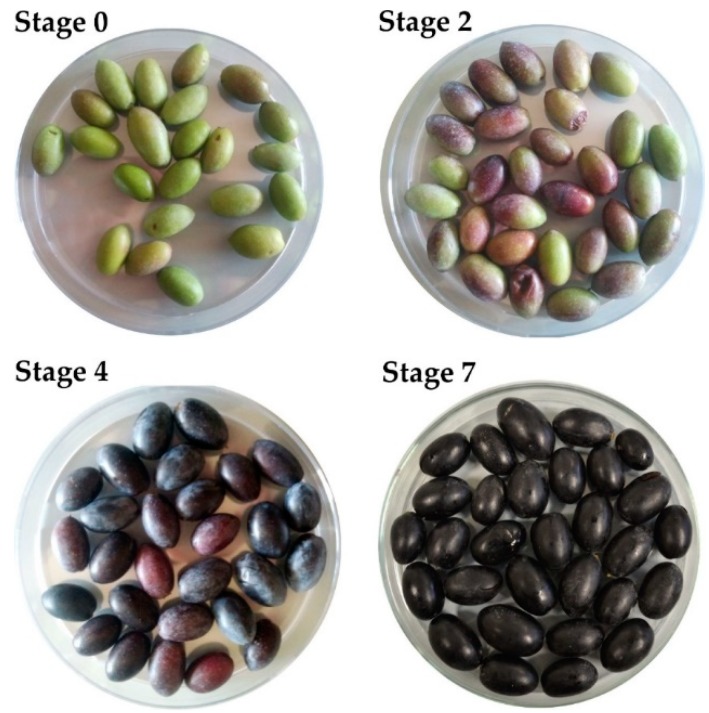
Cellina di Nardò olives. The images represent four of the eight Maturity Index (MI) classification groups (0–7) of olives described by Guzman et al. [16]. Stage 0: green olives; Stage 2: Olive peel partially pigmented and green pulp; Stage 4: dark/black peel and yellow pulp; Stage 7: dark/black peel and pulp.

To evaluate the antioxidant activity and phenolic content during ripening, the olives of CdN cultivar were collected from four of the eight Maturity Index (MI) classification groups (0–7) of olives described by Guzman et al. [16] (Figure 1).

- Green olives, characterized by green peel and light green pulp (MI group 0, indicated as Stage 0);
- Partially green olives with slightly pigmented peel and green pulp (MI group 2, indicated as Stage 2);
- Olives with purple peel and yellow pulp (MI group 4, indicated as Stage 4);
- Black coloration of the peel and pulp identified (MI Group 7, indicated as Stage 7)

Drupes were collected from three different orchards in Salento.

To obtain CdN table olives, fully-ripened olives are traditionally first washed for two days (replacing the water several times) and then deposited in barrels of about 300 kg, covered with saturated brine, and left to ferment naturally (without the addition of chemical products for debittering) until they reach a pH below 4.0; the process takes an average of six months. CdN table olives were analyzed and compared with commercial table olives (Leccino, Blanqueta, Ogliarola, Empeltre, Hojiblanca, Kalamata) purchased on the market. Leccino and Ogliarola table olives were locally fermented essentially as described for CdN olives, whereas table olives of the other cultivars (much more common on the market) were harvested green and darkened by oxidation.

To extract the phenols from edible part of olives, the pulp of 60 olives was homogenized and 5 g were sampled. Then, 50 mL of a cold solution of methanol/water (80/20 *v*/*v*) acidified with HCl (pH 2.5) were added. Methanol and pulp were mixed by continuous agitation for 30 min. The homogenate was then filtered. The pulp was recovered from the filter and mixed again with 50 mL of the same methanol/water solution. A second vacuum filtration was then performed, and the filtrate was added to the previous one. Since the eluates could contain lipid traces, two treatments with an equal volume of hexane (≥97%, HPLC grade) were carried out to remove fatty acids. The extracts were finally concentrated by evaporation under low pressure and cold, avoiding any residues of methanol and obtaining, therefore, aqueous extracts (approximately 10 mL).

### 2.2. Chemicals

Water, methanol, acetonitrile, and hexane were HPLC/MS grade and were provided by Sigma Aldrich (Milan, Italy), as were 6-hydroxy-2,5,7,8-tetramethylchromane-2-carboxylic acid (Trolox), 2,2-diphenyl-1-picrylhydrazyl (DPPH), quinic acid, rutin, verbascoside, quercitrin, luteolin 7 glucoside, luteolin 7 rutinoside, oleuropein, luteolin, quercetin, apigenin 7 glucoside, cyanidin 3 glucoside, and cyanidin 3 rutinoside, which were of analytical standard grade.

### 2.3. Total Phenolic Content (TPC)

Phenols were determined using the Folin–Ciocalteau method described by Singleton and Rossi [17]. Here, 500 μL of olive extract were mixed with 2.5 mL of distilled water and 500 μL of Folin–Ciocalteau reagent. After 4 min, 2 mL of 10% NaCO_3_ and 4.5 mL of distilled water were added. After one hour, the UV-VIS spectrophotometer absorbance was reported (λ = 756 nm).

A calibration curve was calculated using the gallic acid as reference. The calculated equation was:

Optical Density (O.D.) = 4.958X + 0.0437 with *R*^2^ = 0.9997

Results were reported as gallic acid equivalents (mg/g of dried pulp). 

### 2.4. Antioxidant Activity

Antioxidant activity was evaluated using three different assays: the 2,2-diphenyl-1-picrylhydrazyl (DPPH) test, as reported by Goristen et al., [18]; the Oxygen Radical Absorbance Capacity (ORAC) test, as reported by Wang et al., [19]; and superoxide anion scavenging activity analysis, as described by Dasgupta et al. [20]. All the essays were performed in triplicate and the antioxidant activity were expressed as µmol of Trolox equivalent mg^−1^ of dry weight (DW).

### 2.5. HPLC ESI/MS-TOF Analysis of Olive Extracts

The phenolic compounds characterization was performed using Agilent 1200 High Pressure Liquid Chromatography (HPLC) System (Agilent Technologies, Palo Alto, CA, USA) equipped with a standard autosampler, as reported by Nicolì et al. [21]. The HPLC system was coupled to an Agilent diode-array detector (detection wavelength 280 nm) and an Agilent 6320 TOF mass spectrometer equipped with a dual ESI interface (Agilent Technologies) operating in negative ion mode. Detection was carried out within a mass range of 50–1700 *m*/*z*. Accurate mass measurements of each peak from the total ion chromatograms (TICs) were obtained by means of an ISO Pump (Agilent G1310B) using a dual nebulizer ESI source that introduces a low flow (20 μL·min^−1^) of a calibration solution which contains the internal reference masses at *m*/*z* 112.9856, 301.9981, 601.9790, and 1033.9881, in negative ion mode. The identification of anthocyanins was carried out with the same method, but with positive ionization, using the internal reference masses at *m*/*z* 121.050873, 149.02332, 322.048121, and 922.009798.

The quantification of anthocyanins was achieved using calibration curves of authentic chemical standards cyanidin 3 glucoside and cyanidin 3 rutinoside [22], using an HPLC Agilent 1100 coupled with an Agilent DAD sensor (detection wavelength 280 nm and 520 nm). Separation was carried out at 30 °C with a gradient elution program at a flow rate of 0.8 mL/min using a Phenomenex Gemini C18 250 × 4.6 mm, 5-µm separation column. The mobile phases consisted of water plus 7.0% formic acid (A) and water:formic acid:acetonitrile 43:7:50 (B). The following multistep linear gradient was applied: 0 min, 6% B; 15 min, 30% B; 25 min, 50% B; 30 min, 60% B; The injection volume in the HPLC system was 5 μL.

### 2.6. Statistical Analysis

Data were reported as the mean ± SD and four biological replicates were carried out for each sample. Statistical evaluation was conducted by ANOVA, followed by multicomponent Duncan’s test (*p* < 0.05) to discriminate among the mean values. The *R*^2^ correlation coefficients between TPC and the antioxidant activities were also calculated.

## 3. Results

### 3.1. Total Phenolic Content during Maturation and after Fermentation Processes

**Figure 2:**
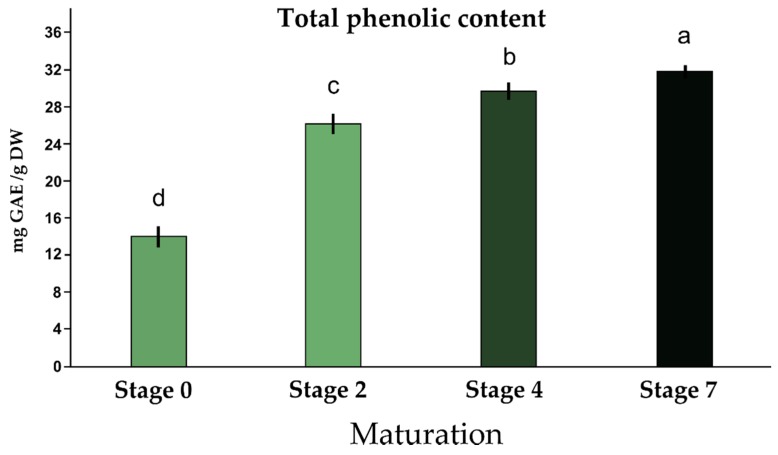
Total phenolic contents (mg GAE/g DW) in Cellina di Nardò olives at four different stages of the maturation process. Results are expressed as mg of GAE/g dried olive pulp. Same letters mean no statistical differences between averages (Duncan test, *n* = 3, *p* = 0.05). GAE: gallic acid equivalent; DW: dry weight.

To evaluate metabolic profiles and antioxidant activities, olives belonging to homogeneous classes of maturity were sampled according to Guzman et al. [16]. Their fresh and dry weight has been determined and the pulp percentage was calculated (data not shown). The TPC during maturation was quantified by the Folin–Ciocolteau method and the results are reported in Figure 2.

As reported in Figure 2, there is a progressive increase in the amount of total phenolic contents during maturation. Green and immature olives, corresponding to Stage 0, have the lowest number of polyphenols, equal to 14.0 mg of gallic acid equivalent (GAE)/g dry weight pulp. In Stage 2, the TPC value increased, reaching 26.18 mg (GAE)/g DW, whereas in Stage 4 TPC was 29.68 mg (GAE)/g DW. When the olives reached full maturity (stage 7), a further increase in total phenolic substances was recorded: 31.80 mg GAE/g DW.

**Figure 3:**
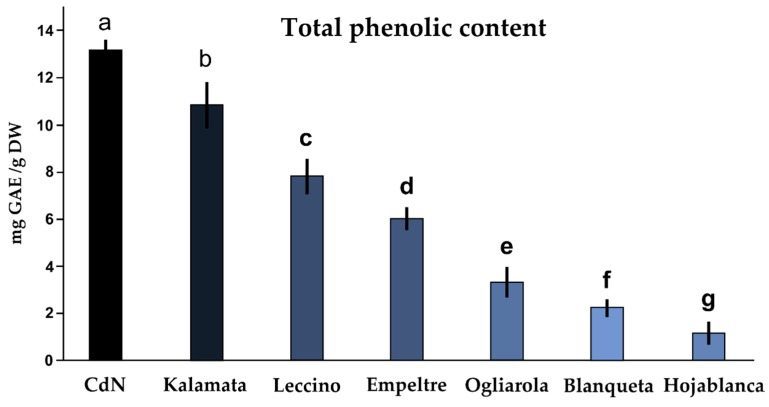
Total phenolic contents (mg GAE/g DW) in seven commercial black table olives. Results are expressed as mg of gallic acid equivalent (GAE)/g dried olive pulp. Same letters indicate no statistical differences between averages (Duncan test, *n* = 3, *p* = 0.05).

Following fermentation/curing, the CdN olives were compared with other six commercial black table olives for the presence of phenolic compounds (Figure 3). It was found that among the seven black table olives, the CdN table olives were the richest in phenolic compounds, with a TPC equal to 13.08 mg/g DW. Since these table olives showed a TPC of 31.80 mg GAE/g DW at full maturity—Stage 7 (Figure 2)—the fermentation process drastically reduced the TPC. Kalamata olives also showed a high content of phenols (10.84 mg GAE/g DW). The lowest level of polyphenols was observed in Hojablanca cultivar (1.19 mg GAE/g DW) (Figure 3).

To identify the principal phenolic compounds in CdN olive extracts, a reverse-phase HPLC/MS-TOF was used. The identification was carried out by comparing the retention times, UV absorbance, and molecular masses with literature data and analytical standard when available.

**Figure 4:**
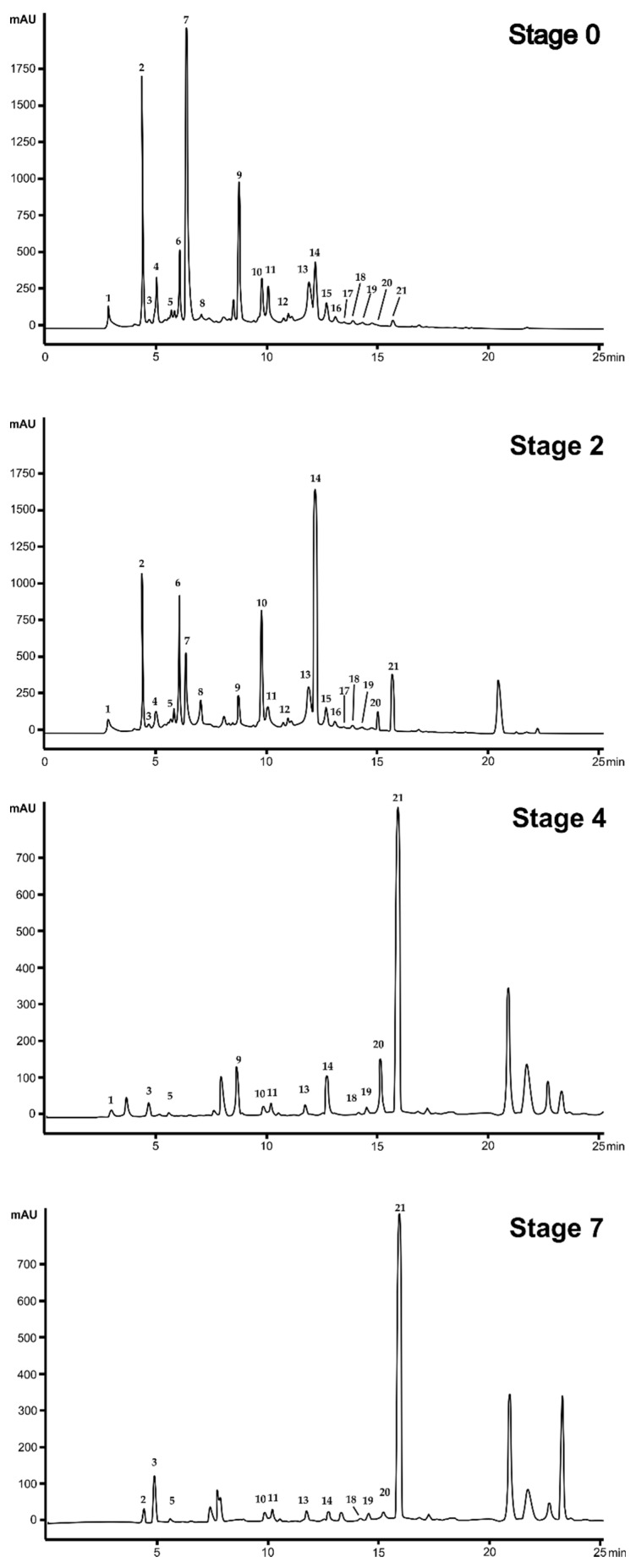
Representative chromatograms of Cellina di Nardò olive extracts during the maturation process. Detection at 280 nm. For the identification of the peaks and relative compounds, see Table 1.

Representative chromatograms of olive extracts during maturation are reported in Figure 4 (A–D) and the list of identified compounds are reported in Table 1.

The metabolic profile during olive maturation (Figure 4) highlighted a great variability among the four analyzed stages. The green olives (Stage 0) had a predominance of substances represented by the peaks 2, 6, 14, 15, and 17, corresponding to hydroxytyrosol glucoside, verbascoside, oleuropein, luteolin, and ligstroside, respectively. During drupe maturation, starting from Stage 4, the quantity of these compounds decreases. Conversely, the concentrations of the anthocyanins cyanidin 3 glucoside and cyanidin 3 rutinoside increase after Stage 4.

### 3.2. Anthocyanin Quantification in CdN Olives

Anthocyanins were identified by 520 nm UV absorbance and molecular weight, and confirmed by authentic chemical standard spectra; the quantification was performed using the calibration curve obtained using the standard cyanidin 3-rutinoside (Table 2).

With the maturation progresses, it is evident that there is a variation of anthocyanin contents: in green fruits (corresponding to Stage 0), there are no anthocyanins; some traces begin to appear in olives belonging to Stage 2; in the olives of Stage 4, anthocyanins are detectable and 3.22 g/kg DW were reported, while fully ripe olives showed 4.62 g/kg DW. The quantity of anthocyanin in fermented CdN olives was lower: 1.16 g/kg DW.

### 3.3. Antioxidant Activity of Olive Extracts

To evaluate the antioxidant properties of the olive fruit extracts during ripening stages, three different antioxidant assays were carried out (ORAC, DPPH, and superoxide anion scavenging activity). Data are reported in Table 3.

As reported in Table 3, the three antioxidant in vitro assays provided similar results among stages. The olive extracts with the highest antioxidant activities were the olives of the Stage 7 (complete maturation). Conversely, the antioxidant activity was lower at earlier maturation stages. However, the ratio and the increase in the antioxidant activity between the olives of Stage 0 and those of Stage 7 were different among the antioxidant assays. The ratio between CdN Stage 0 and CdN Stage 7 in ORAC assay was less than two-fold, whereas in DPPH and superoxide anion assays the ratio was about three-fold. The same analyses were conducted on commercial black table olives, and the results are reported in Table 4.

Whereas the fully ripened CdN olive extracts showed an antioxidant activity of 18,788 ± 3298, 9062 ± 302, and 1.05 ± 0.07, respectively in ORAC, DPPH, and superoxide anion assay, the antioxidant activities of table CdN olives dropped dramatically (an about three-fold reduction) after the fermentation process. However, despite this drastic decrease, the antioxidant activity of CdN olives was the highest among the analyzed table olives. Kalamata and Leccino had similar antioxidant activities of CdN using the DPPH and superoxide anion assays, but the values reported by ORAC assay were statistically different among CdN, Kalamata, and Leccino. Blanqueta and Hojiblanca black table olives showed the lowest antioxidant activities.

## 4. Discussion

Cellina di Nardò is an olive cultivar closely connected to the Salento region by a long cultural tradition. Many plants are over 100 years old, and some of them (the monumental ones) are more than 1000 years old. People of this region have developed an empathic sentiment towards these plants, especially since the *Xylella* epidemic in 2013 [1].

In addition to oil production, CdN is used, in different local food preparations and after appropriate tanning, to produce table olives with a unique and recognizable flavor. Moreover, this olive is harvested when it is completely black (fully ripened), whereas most of the commercial black table olives are darkened by oxidation. The intense black color of the CdN natural black table olive is due to the accumulation in the skin and pulp tissues of a considerable quantity of anthocyanins, pigments that belong to the large family of polyphenols. The anthocyanins, as with polyphenols in general, have considerable beneficial properties as a consequence of their ability to scavenge oxygen radicals. They have good antioxidant, anti-inflammatory, and preventive effects against various pathological states, such as cardiovascular diseases and tumors [29].

The results reported in this work strongly support the possibility to use this table olive as a functional food. We demonstrated that the fully maturation is the best harvest time to obtain a table olive with a high content in phenolic compounds, anthocyanins, and other health valuable compounds. In fact, to assess whether and how the phenolic components vary during maturation, the phenolic content was studied during different ripening stages. The results showed an increase in the total quantity of polyphenols in relation to the stage of maturation; from a value of total phenolic substances of 14.00 mg GAE/g DW (Stage 0) to a value of 31.80 mg of GAE/g DW (Stage 7). Therefore, a two-fold intensification of total phenolic compounds was observed. These data are in accordance with the values reported by other authors [5]. The same authors observed that phenolic fraction in fresh pulp is about the 2% of the total weight of the pulp. We detected a similar value in fully ripened CdN olives where the phenolic fraction represents the 1.6% of the pulp total weight.

The qualitative analysis of phenolic compounds during maturation (Figure 3 and Table 1) demonstrated a great variation of the phenolic composition during ripening. In particular, from Stage 4, olives start to accumulate anthocyanins (cyanidin-3-rutinoside and cyanidin-3-glucoside), so that at Stage 7, the anthocyanin level reached 4.62 g/kg DW. Amiot et al. [9] reported an increase in anthocyanins matching to the progress of fruit ripening. In fact, the anthocyanin biosynthesis starts when the oleuropein decreases as direct result of an increase of the enzymatic activity of phenylalanine-ammonium lyase during drupe maturation [9]. The anthocyanin content found in CdN olives is higher than the quantity of anthocyanins present in most of other commercial olive fruits [28], as reported by a study conducted on several Italian cultivars. The black olives of Frantoio, a cultivar widespread in Tuscany, has a quantity of anthocyanins equal to 1.25 g/kg of fresh pulp; Ciliegino olives have an even lower quantity of phenolic compounds: 0.50 g/kg of fresh pulp [30].

After fermentation process, the level of anthocyanins in CdN decreased to 1.16 g/kg DW, a reduction of about 75% from the freshly harvested olives.

The same trend was observed for the total phenolic content. In fact, after the fermentation process of CdN, we noticed a drop in phenolic content (about 60%) to 13.08 GAE mg/g DW as reported also by Zou and colleagues [31], who observed how fermentation processes can reduce the total phenolic content by 50%. In the Spanish method fermentation for example, as well as in the Greek method, the debittering phase with NaOH causes the higher loss in phenolic compounds, probably due to the polyphenol oxidation under alkaline conditions [32]. During this process the polyphenols undergo to chemical transformations (hydrolysis of oleuropein into hydroxytyrosol) and their concentrations usually decrease. The following processing steps such as lactic fermentation, on the contrary, do not seem to affect total phenolic compound reduction [32].

To produce table olives from the CdN cultivar, completely ripened drupes (Stage 7) are usually employed, determining a high phenolic content after fermentation which is certainly higher than that of the black table olives on the market (Figure 3). The value is even higher than others found in literature concerning black table olive varieties. For example, in the Chondrolia table olives a total amount of phenolic substances of about 5.4 mg GAE/g FW was reported, while in the Amfiss olives the value is even lower and is equal to 2.3 mg GAE/g of fresh pulp [33]. Ben Othman et al. [34] estimated that the total phenolic content in black table olives is about 4–8 mg GAE/g DW. These data suggest the great potential of CdN cultivar to be used as a food reach in phenolic compounds.

The antioxidant capacities of fresh CdN olives were also investigated at four different maturation stages and after the fermentation process. CdN olives showed antioxidant properties (7415 μmol TE/100 g FW) higher than the average of other vegetables. About other fruits, an ORAC value of 4275 μmol TE/100 g fresh weight was reported for the Red Delicious apple [35], and a value of 1837 μmol TE/100 g fresh weight was reported for grapes [36], although the latter fruit is considered rich in antioxidant substances. Moreover, we observed an increase of the antioxidant activity positively correlated to the increase in phenolic substances (*R*^2^ = 0.832, *R*^2^ = 0.756 and *R*^2^ = 0.957, respectively, for ORAC, DPPH, and the superoxide anion test) as already reported in Tunisian olives [37].

## 5. Conclusions

Different treatments (or curing methods) that are necessary to remove the bitterness of the raw olive and to stabilize them to obtain edible table olives, causing a loss in phenolic substances which also results in a loss of anthocyanins and antioxidant activity. However, CdN black table olives were the richest in polyphenols, consequently possessing the best antioxidant activity among the analyzed black table olives and among other black table olives reported in literature [37]. Moreover, it is plausible that regular consumption of CdN table olives can give real returns in terms of prevention of oxidative stress.

## Figures and Tables

**Table 1:** List of chemicalss and anthocyanins putatively identified by High-Performance Liquid Chromatography coupled to Electrospray Ionization Time-of-Flight Mass Spectrometry (HPLC ESI/MS-TOF) following extraction from CdN olive pulp at different stages of maturation.

N.	Compound	RT ^a^ (min)	(M−H)^−^	*m*/*z* Exp ^b^	*m*/*z* Clc ^c^	Diff. (ppm) ^d^	Score ^e^	Ref.^f^
1	* Quinic acid	2.82	C_7_H_11_O_6_	191.0510	191.0561	−5.89	90.44	[23,24,25]
2	Hydroxytyrosol glucoside	4.63	C_14_H_19_O_8_	315.1095	315.1085	−1.26	96.62	[22,23,25]
3	Secologanoside is. 1	4.85	C_16_H_21_O_11_	389.1095	389.1089	−1.11	88.91	[22,23,25]
4	Secologanoside is. 2	4.94	C_16_H_21_O_11_	389.1101	389.1089	−2.62	96.13	[24,26]
5	* Rutin	5.83	C_27_H_29_O_16_	609.1474	609.1461	−2.15	90.20	[24,26]
6	* Verbascoside	6.02	C_29_H_35_O_15_	623.2013	623.1618	−0.05	93.73	[24,25]
7	Elenoic acid glucoside	6.31	C_17_H_23_O_11_	403.1262	403.1246	−3.68	80.90	[24,26]
8	Oleuropein aglycon	7.02	C_16_H_25_O_10_	377.1459	377.1453	−1.23	92.94	[24]
9	* Quercitrin	8.85	C_21_H_19_O1_1_	447.0960	447.0933	−6.05	89.44	[27]
10	Hydroxyoleuropein	9.82	C_25_H_31_O_14_	555.1773	556.1803	−2.04	97.55	[24,27]
11	* Luteolin 7 glucoside is. 1	10.03	C_21_H_19_O_11_	447.0952	447.0933	−3.93	77.64	[24,25]
12	* Luteolin rutinoside	10.95	C_27_H_29_O_15_	593.1517	593.1512	−0.87	97.79	[24]
13	* Luteolin 7 glucoside is. 2	11.87	C_21_H_19_O_11_	447.0948	447.0933	−3.03	96.13	[24,25,26]
14	* Oleuropein	12.21	C_15_H_9_O_13_	539.1772	539.1770	0.03	97.14	[23,24,25,27]
15	* Luteolin	12.53	C_15_H_9_O_6_	285.0419	285.0405	−4.87	97.08	[23,24,25,27]
16	* Quercetin	13.07	C_15_H_9_O_7_	301.0351	301.0354	1.10	96.04	[24,25]
17	Ligstroside	13.88	C_25_H_31_O_12_	523.1823	523.1821	−0.03	97.55	[26]
18	* Apigenin 7 glucoside	14.31	C_15_H_9_O_5_	269.0461	269.0455	−1.77	98.70	[23]
19	Diosmetin	14.72	C_16_H_11_O_6_	299.0566	299.0561	−1.43	98.50	[23]
20	** Cyanidin 3 glucoside	15.03	C_21_H_21_O_11_	449.1081	449.1078	0.66	92.21	[28]
21	** Cyanidin 3 rutinoside	15.82	C_27_H_31_O_15_	595.1658	595.1657	0.16	95.23	[28]

**^a^** RT, Retention time; **^b^** *m*/*z* Exp, mass to charge experimental; **^c^** *m*/*z* Clc, mass to charge calculated; **^d^** Diff., difference between the observed mass and the theoretical mass of the compound (ppm); **^e^** Isotopic abundance distribution match: a measure of the probability that the distribution of isotope abundance ratios calculated for the formula matches the measured data; **^f^** Ref., References. * Confirmed by authentic chemical standard. ** These peaks were identified in positive ion mode (M−H)^+^.

**Table 2:** Anthocyanin contents in Cellina di Nardò olive extracts during maturation stages and after fermentation. Data are reported as g/kg DW of cyanidin 3-rutinoside. Same letters indicate no statistical differences between averages (Duncan test, *n* = 3, *p* = 0.05).

Olive Extract	Cyanidin-3-Rutinoside (g/kg DW)
Stage 0	ND
Stage 2	Traces
Stage 4	3.22 ^b^ ± 0.22
Stage 7	4.62 ^a^ ± 0.06
Table olive (fermented)	1.16 ^c^ ± 0.16

**Table 3:** Antioxidant activity detected in extracts of Cellina di Nardò olives at four different maturation stages. Results are expressed as μmol Trolox Equivalents/100 g FW (ORAC and DPPH tests) and as Inibitory Concentration (IC_50_, μg of FW olive pulp). Same letters indicate no statistical differences between averages (Duncan test, *n* = 3, *p* = 0.05).

Olive Extract	ORAC Test μmol TE/100 g FW	DPPH μmol TE/100 g FW	Superoxide Anion Test IC_50_ (µg FW)
Stage 0	11,412 ^b^ ± 1722	2888 ^d^ ± 234	3.15 ^a^ ± 0.13
Stage 2	13,565 ^b^ ± 2173	4212 ^c^ ± 351	2.15 ^b^ ± 0.35
Stage 4	15,990 ^a,b^ ± 486	6285 ^b^ ± 312	1.45 ^b,c^± 0.49
Stage 7	18,788 ^a^ ± 3298	9062 ^a^ ± 302	1.05 ^c^ ± 0.07

**Table 4:** Antioxidant activity detected in commercial black table olives of seven different cultivars. Results are expressed as μmol of Trolox Equivalents (TE)/100 g FW (ORAC and DPPH tests) and as IC_50_ per μg FW (superoxide anion assay) of olive pulp. Same letters indicate no statistical differences between averages (Duncan test, *n* = 3, *p* = 0.05).

Black Table Olive Extract	ORAC Test μmol TE/100 g FW	DPPH μmol TE/100 g FW	Superoxide Anion Test (IC_50_ µg FW)
Cellina di Nardò	7415 ^a^ ± 353	2920 ^a^ ± 51	4.25 ^c^ ± 0.21
Kalamata	4717 ^b^ ± 96	2533 ^a^ ± 135	6.10 ^c^ ± 0.42
Leccino	3964 ^c^ ± 213	2612 ^a^ ± 686	9.55 ^c^ ± 1.34
Empeltre	2355 ^d^ ± 224	1209 ^b^ ± 247	22.00 ^b^ ± 2.83
Ogliarola	1676 ^e^ ± 87	1186 ^b^ ± 398	22.95 ^b^ ± 2.62
Blanqueta	1537 ^e^ ± 164	1299 ^b^ ± 416	42.21 ^a^ ± 11.60
Hojiblanca	747 ^f^ ± 10	1356 ^b^ ± 246	47.55 ^a^ ± 2.05
